# A tablet computer-based cognitive training program for young children with cognitive impairment: A randomized controlled trial

**DOI:** 10.1097/MD.0000000000019549

**Published:** 2020-03-20

**Authors:** Eun Jae Ko, In Young Sung, Jin Sook Yuk, Dae-Hyun Jang, Gijeong Yun

**Affiliations:** a Department of Physical Medicine and Rehabilitation, Ulsan University Hospital, University of Ulsan College of Medicine, Ulsan; b Department of Rehabilitation Medicine, Asan Medical Center, University of Ulsan College of Medicine; c Department of Rehabilitation Medicine, Asan Medical Center, Seoul; d Department of Rehabilitation, Incheon St. Mary's Hospital, College of Medicine, The Catholic University of Korea, Incheon; e Department of Rehabilitation Medicine, Gamcheon Champyonhan Geriatric Hospital, Pusan, Republic of Korea.

**Keywords:** cognitive impairment, cognitive therapy, early intervention, tablet computer based cognitive training, young children

## Abstract

**Background:**

Evidences suggest that cognitive training facilitates cognitive function, and most studies have targeted adults and children older than 4 years of age. This study investigated the applicability and efficacy of a tablet computer-based cognitive training program for young children with cognitive impairment of cognitive age between 18 and 36 months.

**Methods:**

Thirty-eight children were randomly assigned to the intervention (n = 20, administered a tablet computer-based cognitive training program, for 30 minutes per session and twice a week over a period of 12 weeks) and control (n = 18, received the traditional rehabilitation program) groups. Mental scale of Bayley Scales of Infant Development II (BSID II), Pediatric Evaluation of Disability Inventory (PEDI), interest/persistence domain of the Laboratory Temperament Assessment Battery (LAP-TAB), Early Childhood Behavior Questionnaire (ECBQ), and Goal Attainment Scale (GAS) were evaluated before and after 12 weeks of therapeutic intervention.

**Results:**

The tablet computer-based cognitive training program was applicable to all children in the intervention group without any problems including irritable behavior or obsession about a tablet computer. After 12 weeks, Mental scale of BSID II, PEDI (social function), LAB-TAB (observation), LAB-TAB (manipulation), and GAS showed statistically significant improvements in the intervention group, compared with the values in the control group (*P* < .05). After adjusting for the pre-treatment measurements and cognitive age, the tablet computer-based cognitive training program had significant effect on the post-treatment measurements of Mental scale of BSID II, PEDI (social function), LAB-TAB (observation), LAB-TAB (manipulation), and GAS (*P* *<* .05). There was no association between the change in the scores and the severity of cognitive delay in the most of the measurements, however, the self-care domain of PEDI showed a negative association with the severity of the cognitive delay (r = −0.462, *P* *=* .04).

**Conclusions:**

Application of a tablet computer-based cognitive training program was feasible and showed improvements in cognitive function in young children with cognitive impairment of cognitive age between 18 and 36 months, regardless of the severity of the cognitive delay. But severe cognitive delay can be related with less improvement in the self-care domain of PEDI.

**Trial registration number:**https://cris.nih.go.kr (KCT0002889)

## 1 Introduction

Cognitive impairment is a commonly encountered problem in children with various clinical diseases, including Down syndrome, autism spectrum disorder (ASD), traumatic brain injury, and others. Cognitive impairment is associated with impaired functional outcomes and independent activities of daily living^[1]^ and limits participation in education and society.

Cognitive interventions are not always effective in young children with cognitive impairment.^[2,3]^ Although alternative treatment choices are needed, treatment options are very limited for such children. Computerized cognitive training has become the most popular and accessible form of cognitive training. Evidence suggests that cognitive training facilitates cognitive function, and most studies have targeted adults and children older than 4 years of chronological age.^[4,5]^ As a novel cognitive training program, touch screen technology can easily be applied to very young children and children with a lower cognitive level and can promote motivation due to its ease of use and visual and auditory support. Furthermore, it brings children to pay more attention to the program, resulting in better compliance to the cognitive trainings. Improving attention is very important, because the other cognitive domains including perception-motor function, learning and memory, executive function, and social cognition are basically based on the attention domain of cognitive function.

The tablet computer-based cognitive training program ^[6]^ was developed by a team of pediatric physiatrists, pediatric occupational therapists, pediatric neurologist, pediatric psychiatrist, psychologist, and computer graphic team for young children or individuals with severe cognitive impairment of cognitive age less than 4 years. It is not only the first tablet computer-based cognitive training program but also the first program targeting young children with a cognitive age between 18 and 41 months. Twelve cognitive training programs were designed, of which 6 are adaptive, consisting of 9 or 10 levels with different difficulties, and each level consisted of 10 tasks. If level of difficulty is selected in the program, tasks are provided in random sequence for each trial. These programs were designed to include the basic components of the cognitive domain such as attention, visual and auditory perception, memory, and executive function. There was an evidence that adaptive trainings lead to sustained enhancement of poor working memory in children.^[7]^ The 6 programs that contains different difficulty levels are puzzles, hidden object games, animal matching, pattern matching, identical image identification, and memory games. The other 6 programs are non-adaptive and designed as universal tasks that do not have difficulty levels. They were game-based and were similar to several other previously developed computer-based functional games. These game-type universal tasks were mainly aimed at improving attention span and eye–hand coordination. Furthermore, these tasks were familiar, interesting, and motivational. These 6 non-adaptive programs consisted of tracing, object matching, sound matching, balloon games, farm games, and daily activity games. Each cognitive training program was developed to target on many different cognitive domains including attention, perception-motor function, learning and memory, and executive function, and attention was especially the main target of the training program.

The advantages of this tablet computer-based cognitive training program are as follows:

(1) it is an interactive medium with sounds and animations that increases a child's attention to a given task, resulting in ease of use with young children,
(2) it is based on a tablet computer that uses a touch screen system; therefore, not only children with cognitive impairment but also children with both cognitive and motor impairments could benefit from it,
(3) it could also be applied to older people who have severe cognitive impairment,
(4) its programs are very structured and standardized,
(5) it provides objective feedback, and
(6) it is portable; therefore it can be used anywhere at any time.

The aim of this study was to evaluate the applicability and efficacy of the tablet computer based cognitive training program for young children with cognitive impairment of cognitive age between 18 and 36 months.

## 2 Materials and methods

### 2.1 Study design and participants

**Figure 1:**
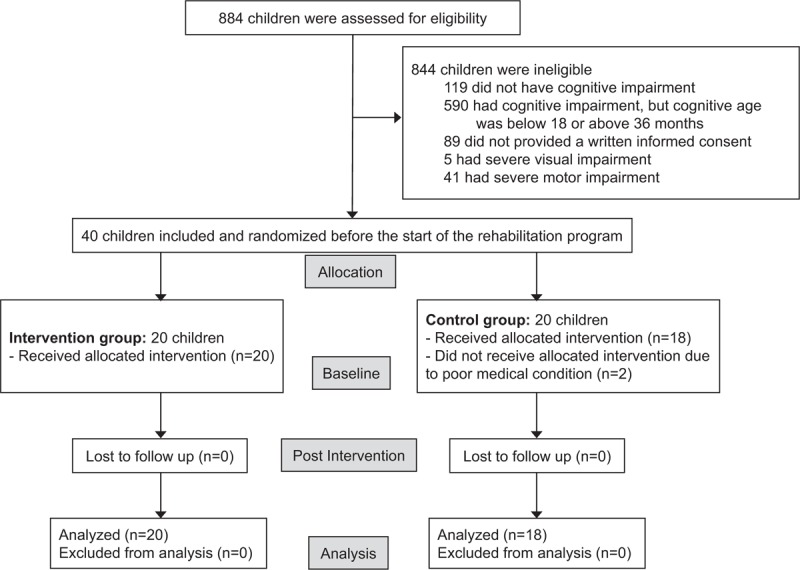
Flow diagram.

This pilot study was approved by the Ethical Committee of Asan Medical Center (ref number: 2016-0091). All main caregivers of the children gave written informed consent before data collection began. The trial has been registered at Clinical Research Information Service (ref number: KCT0002889). Figure 1 shows the flow of patients.

Children who visited the outpatient clinic of the Pediatric Rehabilitation Medicine Division at Asan Medical Center from May 2014 to October 2016 were assessed for inclusion in the study according to the following criteria:

(1) children with cognitive impairment of cognitive age between 18 and 36 months, as assessed with the mental age of the Bayley Scales of Infant Development II (BSID II), and
(2) children whose caregivers provided a written informed consent.

Exclusion criteria were as follows:

(1) children with severe visual impairment (e.g., low vision and blindness) and
(2) children with severe motor impairment (e.g., excessive weakness and increased muscle tone) who could not reach the screen of the tablet computer-based cognitive training program with their hands.

### 2.2 Randomization and masking

Children were randomized according to a computer-generated random number list and allocated to either an intervention group (receiving a tablet computer-based cognitive training program) or control group (receiving a traditional rehabilitation program). A different person from the ones doing the recruitment and providing intervention carried out the randomization, using an online randomization program with a ratio of 1:1. All children and their caregivers, who rated one of the measurement, Early Childhood Behavior Questionnaire (ECBQ), were aware of their treatment allocation in the study design. However, the 2 fixed occupational therapists involved in the evaluation and the 2 investigators who conducted the study were blinded to the treatment allocation.

### 2.3 Intervention

The intervention group was administered the tablet computer-based cognitive training program for 30 minutes per session and twice a week over a period of 12 weeks by occupational therapists in the hospital. At least 1 adaptive program and 1 non-adaptive program were included in 1 session to maximize the efficacy of the program. The duration and frequency of the program was determined based on the previous study by Bennett et al.^[8]^ The occupational therapists helped the children to use the program all the time, and the children interacted with the program for all 30 minutes. Children in the intervention group had different cognitive ability, and the level of difficulty was selected by the occupational therapists. The activities increased in complexity with increased success, with the modulation by the occupational therapists. Therefore, each child had different levels of success with the activities. Children were not allowed to play the same program throughout each session.

In contrast, the control group received the traditional rehabilitation program by occupational therapists for 30 minutes per session. The total number and duration of the sessions were the same in both groups. It consisted of 10 tasks, which included both adaptive and non-adaptive trainings. It included cognitive training (using puzzles, blocks, toy, color matching, identical image identification, finding hidden objects, and tracing), which targeted on attention, perception-motor function, memory, and executive function, and training of activities of daily living (using scissors, pens, and putting on and off the clothes).

### 2.4 Measurements

Children were evaluated by experienced occupational therapists before and after receiving therapeutic intervention for 12 weeks, and the evaluations were done in 7 days before and after the intervention. These 2 fixed occupational therapists involved in the evaluations were not the same occupational therapists involved in the delivery of the intervention program. Cognitive function was assessed using psychodevelopmental and functional measurement scales. The primary outcome was assessed using the Mental scale of the BSID II,^[9]^ and secondary outcomes were: Pediatric Evaluation of Disability Inventory (PEDI),^[10]^ Laboratory Temperament Assessment Battery (LAP-TAB),^[11]^ ECBQ,^[12]^ and Goal Attainment Scale (GAS).^[13]^ BSID II^[9]^ is the most widely used measure to assess developmental progress in Mental and Motor scales between 1 and 42 months of chronological age, however, it is also applicable to children over 42 months of chronological age with developmental delay. Only Mental scale of the BSID II was used in this study. Cognitive age was calculated with the raw mental score of BSID II using a table in the manual of BSID II with a title of “Raw score equivalents for developmental ages for the mental and motor scales.” When the difference between the cognitive age and the chronological age is below 6 months, we considered that there was no cognitive impairment. On the other hand, when the difference between the cognitive age and the chronological age is over 6 months, we considered that there was cognitive impairment. PEDI^[10]^ is an instrument that measures independence in daily living and covers essential daily activities in self-care, mobility, and social functioning in children between 6 months and 7.5 years of age. LAB-TAB^[11]^ assesses infant responses to stimuli that elicit emotional or behavioral reactivity. Only one dimension of “interest/persistence” was used to assess attention, which is assessed with either a block play paradigm^[14]^ or a bead play paradigm,^[15]^ according to the ability of the child. GAS is a criterion-referenced measure of an individual's goal achievement that uses a collaborative process involving an interview between a clinician, child, and parent. It was first introduced by Kiresuk and Sherman^[16]^ in the form of a 5-point scale, but the version used in this study was a 6-point scale introduced by Steenbeek et al.^[13]^ ECBQ^[12]^ is a parent-report measure that evaluates behavior during early childhood between the age of 18 and 36 months. It originally measures 18 discrete traits, but only the “attentional focusing” and “attentional shifting” traits were used in this study as a measurement of attention. All the scores used in this study were raw scores. In addition, data on chronological age, sex, and diagnosis of the children were collected.

### 2.5 Statistical analysis

Data were analyzed using SPSS for Windows version 20.0 (SPSS Inc, Chicago, IL), and mean and standard deviation were obtained with a threshold for statistical significance set at *P* < .05. Normality tests were used before going on analysis. If the variables were normally distributed, parametric statistics were used. If the variables were not normally distributed, nonparametric statistics were used. To compare the baseline characteristics of the two groups, the Independent *t* test, Mann–Whitney *U* test and Chi-square test were used. The Wilcoxon signed rank test was used to compare the pre- and post-treatment measurements of the 2 groups, and the Mann–Whitney *U* test was used to compare the change values of measurements between the 2 groups. The linear regression analysis was used to show the regression of post-treatment measurements on group to adjust for the pre-treatment measurements and cognitive age. To evaluate the efficacy of a tablet computer-based cognitive training program according to the severity of the cognitive delay, Pearson correlation analysis was used in the intervention group. The severity of cognitive delay was defined as the difference between the chronological and the cognitive ages.

## 3 Results

### 3.1 Baseline characteristics of children with cognitive impairment and applicability of the tablet computer-based cognitive training program

Twenty children in the intervention group and 18 in the control group were analyzed. Baseline characteristics of the intervention and control group are given in Table 1. The mean chronological age of the intervention group was 54.8 ± 21.0 months (median 48.5, range 27–91), with 11 males and 9 females, and the mean chronological age of the control group was 51.1 ± 21.7 months (median 46, range 24–109), with 7 males and 11 females. When comparing the baseline characteristics, there was no significant difference between the two groups in the measurements of cognitive function.

**Table 1:**
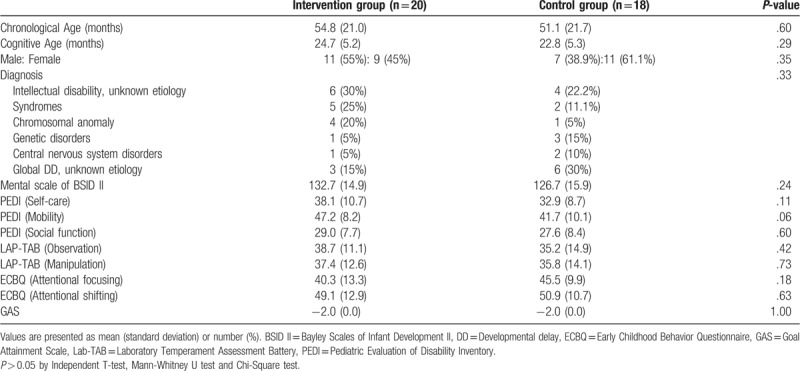
Baseline characteristics of intervention and control groups.

Twenty children in the intervention group with cognitive age between 18 and 36 months were interested in the tablet computer-based cognitive training program, and it was applicable to all of them. They completed the 12-week intervention without any problems including irritable behavior or obsession about a tablet computer.

### 3.2 Comparison of the outcome measurements within the two groups

Table 2 shows the comparison of outcome measurements within the 2 groups. After 12 weeks of treatment, both groups showed significant improvements in all measurements (*P* *<* .05), which suggest that both the tablet computer-based cognitive training program and the traditional rehabilitation program resulted in the improvements of cognitive function.

**Table 2:**
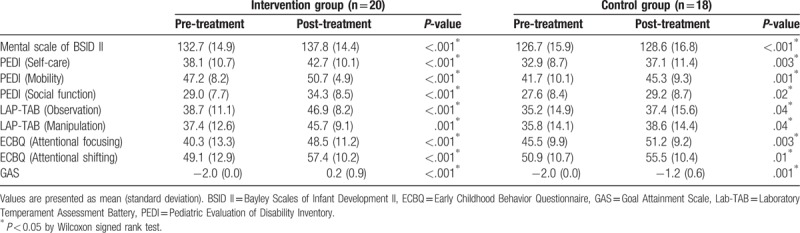
Measurement at pre-and post-treatment in intervention and control groups.

### 3.3 Comparison of the outcome measurements between the two groups

When comparing change values, the intervention group showed more improvements than the control group in most of the measurements (Table 3). Among them, Mental scale of BSID II, PEDI (social function), LAB-TAB (observation), LAB-TAB (manipulation), and GAS showed statistically significant improvements in the intervention group, compared with the values in the control group (*P* < .05).

**Table 3:**
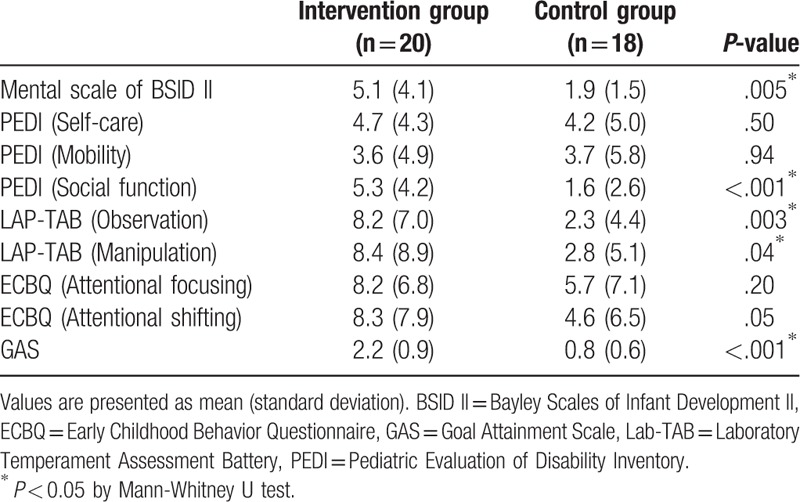
Comparison of change values in measurements between the two groups.

Since baseline functioning and cognitive age were relatively higher for the intervention group compared to the control group (Table 1), we adjusted for pre-treatment measurements and cognitive age by including them as covariates in the linear regression analysis. Table 4 shows that the tablet computer-based cognitive training program had significant effect on the post-treatment measurements of Mental scale of BSID II (Beta = −3.336; *P* = .004), PEDI (social function) (Beta = −3.505; *P* = .01), LAB-TAB (observation) (Beta = −6.836; *P* = .001), LAB-TAB (manipulation) (Beta = −6.051; *P* = .01), and GAS (Beta = −1.242; *P* *<* .001) after the pre-treatment measurements and cognitive age were adjusted for.

**Table 4:**
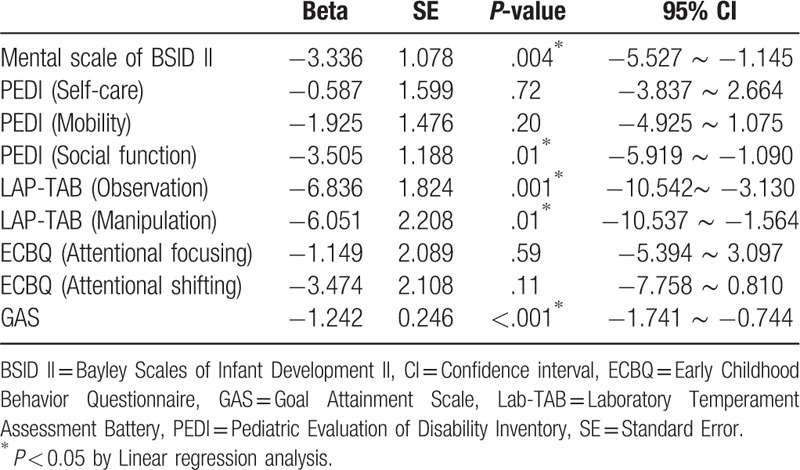
The impact of the tablet computer-based cognitive training program on the post-treatment measurements after adjusting for the pre-treatment measurements and cognitive age.

### 3.4 The efficacy of the tablet computer-based cognitive training program according to the severity of the cognitive delay

Table 5 demonstrates that there was no association between the change in the scores and the severity of cognitive delay in the most of the measurements. However, the self-care domain of PEDI showed a negative association with the severity of the cognitive delay (*r* = −0.462, *P* *=* .04).

**Table 5:**
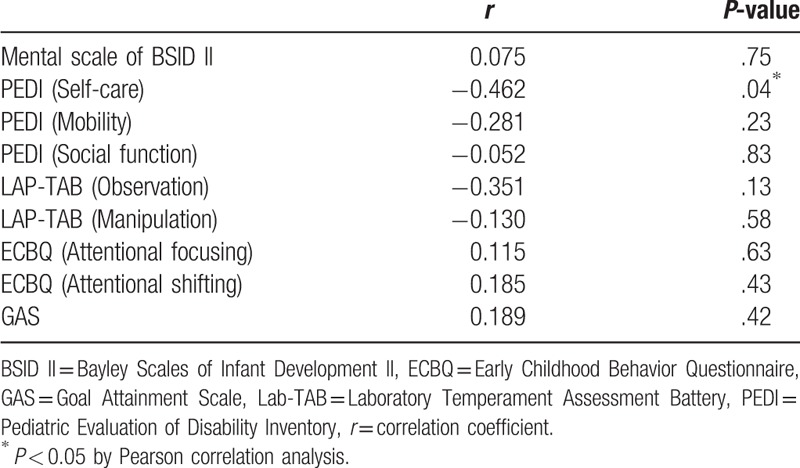
Pearson correlation analysis between the change in the scores and the severity of cognitive delay in the intervention group (n = 20).

## 4 Discussion

The results of this study indicate that the tablet computer-based cognitive training program for 30 minutes per session and twice a week over a period of 12 weeks significantly improved cognitive function, as proven by scores on Mental scale of BSID II. Improvement of Interest/persistence domain of LAB-TAB including duration of observation and manipulation signifies the improvement of attention, which was the main targeting cognitive domain of the tablet computer-based cognitive training program. GAS which indicates an individual's goal achievement also showed significant improvement. In the measurement of PEDI, only social function, but not self-care or mobility domain, showed significant improvement after the tablet computer-based cognitive training program, and it is thought as a result by the improvement of cognitive function. Furthermore, attentional focusing and attentional shifting of ECBQ also showed more improvement in the intervention group than the control group, however it was not statistically significant. When the efficacy of the tablet computer-based cognitive training program was evaluated according to the severity of the cognitive delay, there was no association between the change in the scores and the severity of cognitive delay in the most of the measurements, indicating that the improvement of the scores are not affected by the severity of cognitive delay. However, the self-care domain of PEDI showed a negative association with the severity of the cognitive delay (*r* = −0.462, *P* *=* .04), indicating that severe cognitive delay is related with less improvement in the self-care domain of PEDI. These findings support that the tablet computer-based cognitive training program is effective in the improvement of cognitive function in young children with cognitive impairment of cognitive age between 18 and 36 months, regardless of the severity of the cognitive delay. But severe cognitive delay can be related with less improvement in the self-care domain of PEDI, and this result might be related with the hypothesis that minimum cognitive capacity or training ability may be necessary for the cognitive training to be beneficial.^[17]^

The control group in this study was active control group, who had a similar training schedule to the intervention group. The control group received the traditional rehabilitation program by occupational therapists, which included both adaptive and non-adaptive trainings, and the intervention group received a tablet computer-based cognitive training program, which also included both adaptive and non-adaptive trainings. Therefore, the result of this study could not conclude which type of training (adaptive or non-adaptive) actually brought improvements in measurements. It could only conclude that a tablet computer based cognitive training program including adaptive and non-adaptive trainings was more effective in improving cognitive function than the traditional rehabilitation program including adaptive and non-adaptive trainings in children with cognitive impairment.

It was generally believed that cognition is a fixed individual trait and as such cognitive rehabilitation therapy is not effective in children with cognitive impairment. However, studies during the 2000s reported that cognitive function in children with cognitive impairment could be improved by cognitive rehabilitation programs.^[18,19]^ This concept is based on the theory articulated by Feuerstein et al known as “cognitive modifiability” and “learning experience”.^[20]^ There are research studies that confirm the presence of neuroplasticity, which is the ability of brain structures to change, in pediatric rehabilitation.^[21,22]^ Therefore, early intervention through cognitive rehabilitation programs is important because their effects can vary according to the chronological age of the child. Furthermore, considering the fact that a child's brain between birth and 3 years of chronological age is developing most rapidly, appropriate experiences, environments and cognitive intervention during this period are very important.

Cognitive rehabilitation intervention methods include traditional cognitive rehabilitation programs administered by occupational therapists and computer-based cognitive rehabilitation programs. Computer-based cognitive rehabilitation programs are novel, emerging technology, originally developed by Glisky et al in 1986 for memory training.^[23]^ Nowadays, these programs are popular and accessible form of cognitive rehabilitation intervention and offer very structured and standardized tasks that enhance attention, concentration, memory, and perception-motor skills. The advantages of these programs are that their degree of difficulty can be adjusted according to the person's cognitive level, and they provide prompt, objective feedback that minimizes subjective intervention by therapists. Furthermore, information on performance is stored in a database, allowing systematic management of cognitive rehabilitation, which is very efficient.

Most computer-based cognitive rehabilitation programs currently target adults, and there are many studies that have demonstrated their efficacy of it in adults with acute stroke,^[24,25]^ dementia,^[26]^ and elderly adults.^[27,28]^ However, evidence for the efficacy of these programs are not generalized in pediatric population, because it is very difficult to develop and verify the efficacy of the programs for children. Therefore, only a few types of computer-based programs for children are currently in use. The following are the most widely used programs at present. CogMed is a working memory training program that targets adults and children above 4 years and older. The program has shown improvements in working memory capacity following training for 5 days per week, over a period of 5 to 6 weeks. It has been used for children and adults with attention deficits,^[29]^ intellectual disabilities,^[17]^ learning disorders,^[30]^ and traumatic brain injury or stroke,^[31,32]^ and adults experiencing information overload or the natural effects of aging.^[33]^ In a study with children with intellectual disabilities,^[17]^ 5 weeks of adaptive training program helped improve their cognitive performance in contrast to the active control group (non-adaptive version of the program). Fast ForWord-Language is another computer-based intervention program designed to improve oral language and literacy skills in children with language learning weaknesses. The program was developed based on the theory that language and literacy learning difficulties in children may arise from impairments in rapid auditory temporal processing skills. It targets children with language difficulties between 4 and 14 years of chronological age. The developers of Fast ForWord-Language assert that the program leads to neural reorganization, resulting in increased ability to perceive fast changing acoustic input and subsequent gains of 1 to 1.5 years on standardized tests of language skills after 4 weeks of training.^[34,35]^ Fast ForWord-Language was launched commercially in 1997 and is used in many schools and clinics in the United States, Canada, and Australia as well as in the United Kingdom and other countries. However, there has also been a study that asserted the benefits of Fast ForWord-Language are barely superior to conventional language interventions,^[36]^ and a meta-analysis indicated that the groups administered Fast ForWord-Language showed no significant effect on a variety of outcome measures compared with active or untreated control groups.^[37]^ Timocco is an online, therapeutic gaming environment particularly designed for children with special needs, which targets a range of different populations, including cerebral palsy, ASD, attention deficit hyperactivity disorder (ADHD), learning disabilities, and developmental coordination disorder. The contents and graphics of the game environment appeal to the sensibilities of young children from 3 to 8 years of chronological age. A case study of a 5-year-old child with developmental coordination disorder suggested that the game improved motivation to cope with motor and cognitive challenges and resulted in attempting to obtain new experiences outside the virtual environment in daily living.^[38]^ The Training Attention and Learning Initiative (TALI) is a computerized training program that targets attention skills via four activities delivered on a touch screen tablet. In recent study, 5 weeks of TALI in children with intellectual and developmental disabilities (n = 76; IQ < 75) aged 4 to 11 years enhanced selected attention, compared to the non-adaptive control group.^[39]^

Despite increasing interest in and use of computer-based cognitive programs, evidence for their efficacy is insufficient according to a review.^[4,5]^ In a systematic review of a computer-based cognitive training for ADHD,^[4]^ 16 previous studies were included, of which 14 were randomized controlled trials. The studies included had different types of training (adaptive inhibitory training, adaptive working memory training, adaptive attention training, and adaptive executive function graining) using different programs (RoboMemo, Captain's Log, Brain Train, and Pay Attention), different training duration (between 25 days and 13 weeks), and different types of controls (non-adaptive training group, treatment as usual, and waiting list). It concluded that the effects on underlying ADHD-related neuropsychological deficits might be more consistent but seemed to be limited to near-transfer effects. There was another recent review of the effect of cognitive trainings in children with intellectual disabilities.^[5]^ Seventeen studies were included for working memory training studies and they had different chronological age of patients (between 4 and 15 years), different programs (Jungle Memory, CogMed working memory training, Mate Marote, Odd Yellow), different training sessions (between 5 and 28 sessions), different training duration (between 2 and 14 weeks), and different types of controls (active and passive). Ten studies were included for attention training studies and they had different chronological age of patients (between 11 months and 12 years) using different programs [Training executive, attention and motor skills (TEAMS), Pay Attention, Task Switching, Braintrain, Computerized progressive attentional training program (CPAT)], different training sessions (between 7 and 84 sessions), different training duration (between 4 and 12 weeks), and different types of controls (active and passive). It concluded that cognitive training programs that focus on domains such as attention and working memory, have shown some promising results, however there is insufficient evidence to truly evaluate the efficacy of such interventions.

Although there are several computer-based programs for children, they currently target older children, and until now there have been no computer-based programs targeting children younger than 4 years of cognitive age and people with severe cognitive impairment. This randomized controlled trial verified the applicability and efficacy of the tablet computer-based cognitive training program in young children with cognitive impairment with cognitive age between 18 and 36 months. The results of this study provide objective and scientific evidence for the use of the program as an early intervention in cognitive rehabilitation. If effective early intervention in cognitive rehabilitation is provided to children with cognitive impairment, there would be resultant improvements in cognitive function, social adaptation, and active social participation in the society.

## 5 Limitations

This study has some limitations. First, a small number of children from only one organization were included. Second, although PEDI is an instrument that measures independence in daily living, it has a limitation in evaluating daily activities in real life. Third, the measurements used in this study were primarily focused on attention because other domains of cognitive function, such as memory or executive function are difficult to evaluate in children with cognitive impairments. Fourth, only objective measurements were evaluated, and subjective satisfaction of the main caregivers were not assessed. Fifth, there were no objective measurements or questionnaires to evaluate the degree of the interest toward the program and the problems associated with it. Based on the interviews with caregivers and therapists who provided the program, the fact that children were interested in the program and there was no problems including irritable behavior or obsessions about a tablet computer during and after the study were deduced. Sixth, the differences in training progress or performance can affect training outcomes, however, this information was not recorded in this study. Therefore, it could not be accounted in analyzing the result. Seventh, there was a lack of a true negative control group in this study, in which children do not attend any programs. Since it is unethical for children to stop the rehabilitation program they were attending, a true negative control group was absent. Lastly, there was no long-term follow-up assessment to evaluate retention effect. Further testing with a larger sample population is needed to study the lasting effects and real-world application of cognitive training.

## 6 Conclusions

Application of a tablet computer-based cognitive training program was feasible and showed improvements in cognitive function in young children with cognitive impairment of cognitive age between 18 and 36 months, regardless of the severity of the cognitive delay. But severe cognitive delay can be related with less improvement in the self-care domain of PEDI. This tablet computer-based cognitive training program is a portable touch screen system with structured and standardized program and provide interaction with children using sounds and animations that increases children's attention. Since it is very difficult to administer treatments to young children with cognitive impairment, we believe that this study is able to give another option of cognitive rehabilitation intervention for them.

## Acknowledgments

The authors thank the statistician, Eun Ji Park (Medical Information Center, Ulsan University Hospital) who helped us with statistics. The authors also would like to thank all the participants.

## Author contributions

**Conceptualization**: Eun Jae Ko, In Young Sung, Jin Sook Yuk, Dae-Hyun Jang, Gijeong Yun.

**Data curation**: Jin Sook Yuk, Gijeong Yun.

**Formal analysis**: Eun Jae Ko, Dae-Hyun Jang.

**Investigation**: Eun Jae Ko, In Young Sung.

**Writing – original draft**: Eun Jae Ko.

**Writing – review & editing**: Eun Jae Ko, In Young Sung, Jin Sook Yuk, Dae-Hyun Jang, Gijeong Yun.

In Young Sung orcid: 0000-0001-6545-6744.
